# Bringing Technology to Justice-Involved Youth

**DOI:** 10.1007/s10802-026-01428-z

**Published:** 2026-02-21

**Authors:** Esther C. A. Mertens, J. J. Asscher, C.-S. Sergiou, J.-L. van Gelder

**Affiliations:** 1 Department of Criminology, Max Planck Institute for the Study of Crime, Security and Law, Freiburg im Breisgau, Germany; 2 Netherlands Institute for the Study of Crime and Law Enforcement, Amsterdam, the Netherlands; 3 Forensic Child and Youth Care Sciences, Utrecht University, Utrecht, the Netherlands; 4 Department of Child and Adolescent Psychiatry and Psychosocial Care, Amsterdam University Medical Center, Amsterdam, the Netherlands; 5 Amsterdam Public Health, Mental Health, Amsterdam, the Netherlands; 6 Institute of Education and Child Studies, Leiden University, Leiden, the Netherlands

## Abstract

Conventional assessment and treatment approaches targeting justice-involved youth have typically yielded low engagement and modest long-term impact on recidivism and psychosocial functioning. Technologies such as virtual reality, smartphone applications, and wearable devices, offer promising opportunities to address such limitations by providing scalable, engaging and ecologically valid approaches that align well with the often complex needs of this population. The contributions in the special issue aim to showcase some of the potential of these technologies. The special issue originated from a 5-day international Lorentz workshop that brought together researchers and professionals working in clinical practice to explore opportunities, needs, and barriers related to technology implementation in this context. The included contributions comprise empirical case studies illustrating efficiency gains through technological support of existing practices; papers detailing innovative methods that leverage unique capabilities of specific technologies, such as enhanced accessibility and immersive experiences; state-of-the-art reviews; and a viewpoint paper addressing ethical considerations. Collectively, the contributions highlight the promise of technology as well as the need for methodologically robust research to ensure effective and ethical implementation.

## Introduction

Despite relatively stable and even declining rates in recent years, juvenile delinquency remains a societal concern (Tollenaar & Van der Laan, 2023). While effective assessment methods and treatments are essential to further reduce juvenile delinquency, the effectiveness of current approaches is generally modest (Pappas & Dent, 2021). We argue that technological innovations hold significant potential to improve prevention, assessment, and rehabilitation efforts, thereby ultimately contributing to reduced reoffending rates among justice-involved youth. Such innovations offer unique opportunities –such as 24/7 availability, gamification, and immersive experiences– that align well with the diverse and complex needs of this population. To explore this potential, the contributions to this special issue investigate the scientific underpinnings of technology-based innovations in the assessment and treatment of justice-involved youth.

This special issue stems from an international Lorentz workshop on the use of technology in assessment and treatment for justice-involved youth. The workshop was held at the Lorentz Center in Leiden, the Netherlands, from 13 to 17 January 2025, and was organized in collaboration with the Max Planck Institute for the Study of Crime, Security and Law. The impetus for the workshop arose from the observation that despite the vast potential technology offers to enhance existing intervention methods, it is currently underused in the juvenile justice system. To address this gap, the workshop aimed to stimulate research on innovative applications of technology in this context, with the ultimate goal of enabling justice-involved youth to benefit from technological advancements. By bringing researchers and professionals working in clinical practice together, the workshop provided a platform to explore needs and preferences and identify barriers related to integrating technology into this domain. Participants were invited to submit papers presenting scientific overviews or empirical case studies on the current state of technology use –specifically Virtual Reality (VR), smartphone applications (apps), and wearables– within the juvenile justice setting.

### Assessment and Treatment for Justice-Involved Youth

Justice-involved youth are a heterogeneous group of young people (typically aged 12 to 23) who have become involved in the juvenile justice system, resulting in adjudication, incarceration, or participation in probation and diversion programs. These youth are treated as a special group by the criminal justice system, because the rehabilitative perspective generally prevails over the punishment goal, aligning with the principles of the international Convention on the Rights of the Child. These principles oblige governments to implement intervention initiatives aimed at reducing the risk of youth reoffending. Compared to their peers in the general population, justice-involved youth are characterized by a higher prevalence of complex mental health problems (both externalizing and internalizing), academic problems, substance use, and traumas (e.g., Burke et al., 2015; Dierkhising et al., 2013). Pressing needs include reliable behavioral and mental health assessment as well as effective treatment.

Assessments help to identify risk and protective factors, set intervention goals, and guide decisions about probation and reintegration (Leach & Powell, 2020). Furthermore, treatments may play a role in successful rehabilitation and reintegration into society, thereby reducing recidivism. Adequate diagnostics and treatment of justice-involved youth are complicated due to specific characteristics of this group, such as lower cognitive abilities and literacy, as well as the generally low motivation of these youth for (court-mandated) assessment and treatment (King et al., 2023; Leach & Powell, 2020). Additionally, there can be systematic barriers to receiving appropriate assessment and treatment such as freedom restrictions, lack of resources, limited availability, difficulties in identifying appropriate programs, and high costs (Johnson-Kwochka et al., 2022). These individual and systemic aspects together make the assessment and provision of effective treatment for justice-involved youth particularly challenging, as indicated by the generally small effects of intervention efforts among this population (Pappas & Dent, 2021).

Leveraging technology to enhance assessment and treatment strategies for justice-involved youth could provide the innovation necessary to improve intervention outcomes within the juvenile justice system. Employing technology can enhance accessibility to behavioral and mental health care. It can support, offer alternatives, or provide ‘add-on’ components to in-person intervention (Stoll et al., 2020); it can also facilitate personalization of content (Stoll et al., 2020), enabling enhanced adaptation to the needs of individual youth. It has already been successfully applied in various types of intervention, including assessment, psychoeducation, psychotherapy, pharmacotherapy, crisis intervention, and counseling (Kirschstein et al., 2023). Initiatives that apply technology specifically for adults in the criminal justice setting have shown promise (e.g., Kip et al., 2018). As “digital natives”, youth may be even more inclined to accept and feel comfortable with using technology. But even though these youth indicate a preference for behavioral and mental health interventions that incorporate technology (Klymkiw et al., 2024), its use for intervention purposes among justice-involved youth is still limited (e.g., Grove et al., 2021).

### The Potential of Technology

Broadly speaking, technology can be used to innovate assessment and treatment in at least two ways: (1) through improving efficiency, and (2) through bringing innovative methods. Concerning efficiency, technology can enhance intervention efforts by taking over certain tasks or elements of existing methods. Applying technology for straightforward tasks can reduce clinicians’ workload, leaving more time for them to focus on intervention aspects that require their expertise. For example, instead of a clinician providing psychoeducation, a smartphone app can be used (Kirschstein et al., 2023). Regarding innovative methods, technologies may offer unique possibilities that are difficult or even impossible to achieve using conventional methods. To illustrate, VR enables people to embody avatars with characteristics that differ from their own, and digital interventions allow for immediate and continuous access to treatment content through an app or website. Of course, ‘efficiency’ and ‘innovation’ are not mutually exclusive as innovations aiming to support existing methods can also leverage the technology’s unique possibilities and vice versa.

The current special issue presents papers that leverage both of these possibilities (see for the online collection https://link.springer.com/collections/gbheigheah). The issue contains empirical case examples of ‘efficiency’, in which technology is used to support existing assessment and treatment methods (Helseth et al., 2025; Folk et al., 2025; Klein Schaarsberg et al., 2025). It also includes examples of ‘innovative methods’ that showcase how the unique possibilities of technology can be leveraged to innovate assessment (Van Berkel et al., 2025; Westerveld et al., 2025).

Furthermore, resulting from discussions during the workshop, the special issue includes overviews of state-of-the-art technology-based assessment and treatment initiatives (VR: Mertens & Van Gelder, 2025; Smartphone apps: Aarts et al., 2025; Wearables: Bergwerff et al., 2025), and a viewpoint paper on ethical considerations when employing technology for assessment and treatment in the juvenile justice setting (Kip et al., 2025).

#### Efficiency: Technology in Support of Existing Assessment and Treatment Methods

Enhanced efficiency by using technology in support of conventional methods can be achieved in various ways. For instance, Helseth et al. (2025) used technology to increase accessibility to behavioral health care by developing an ‘add-on’ component to usual services. They developed an app to reduce cannabis use among justice-involved youth who diverted from the formal juvenile legal system. This app was designed to overcome several barriers these youth face to receiving services in the community. Folk et al. (2025) used technology to expand accessibility to mental health care for caregivers of detained adolescents, as particularly this group of caregivers has limited access to behavioral and mental health services. Together with the target group, they co-designed and pilot-tested an app aiming to reduce parenting stress among caregivers of detained adolescents.

Klein Schaarsberg et al. (2025) provide an example of how technology can support in-person treatment for adolescents with disruptive behavior problems. The treatment was aimed at decreasing cognitive distortions and increasing treatment motivation via mentalization. During treatment sessions, adolescents engaged in perspective-taking exercises supported by technology (i.e., VR) that afforded the visualization of the discussed scenarios.

#### Innovative Methods: Unique Possibilities of Technology for Assessment and Treatment

Besides supporting clinicians, technology also offers possibilities that are difficult, unethical, or even impossible to achieve through conventional assessment and treatment methods. Some of the possibilities –or ‘affordances’– of VR in the context of assessment and treatment are described in the scoping review by Mertens and Van Gelder (2025). The authors highlight six VR affordances and illustrate their usefulness with examples from VR-based assessments and treatments implemented in the forensic setting. Among other things, the authors point out VR’s ability to mitigate challenges due to freedom restrictions and the possibility to elicit real-world behavior under safe and controlled circumstances, making this technology particularly suited for intervention purposes in this setting.

Case examples of how the unique possibilities of VR can be leveraged for assessment in the forensic setting are presented by Van Berkel et al. (2025) and Westerveld et al. (2025). Van Berkel et al. (2025) assessed sibling aggression by virtually transporting young adolescents to simulated environments. In these environments, the adolescents could verbally and physically interact with their ‘sibling’ –the sibling-avatar was actually controlled by a researcher who sought to provoke anger and aggression. The observed aggressive behavior in VR was related to self-reported aggression but not to aggression reported by siblings and parents. In a similar vein, Westerveld et al. (2025) immersed detained adolescents in a virtual playground to assess their aggression. This assessment was conducted during pre-trial detention, a moment in which it is usually difficult to assess aggression in a reliable and valid way as there is generally a tendency towards social desirability. While on the virtual playground, adolescents played a game on a virtual tablet and were provoked by clinician-controlled avatars to elicit aggression. Though the observed behavior in VR showed little convergence with self-reports, it did predict real-life violent institutional infractions.

The unique possibilities of apps for intervention purposes in the criminal justice setting are outlined in the scoping review by Aarts et al. (2025). The authors emphasize the ability of apps to deliver immediate, tailored, and accessible support that can be used anytime and anywhere with simple content and easy-to-navigate interfaces. Additionally, apps can utilize the sensors integrated into smartphones for assessment and treatment purposes, for instance, to deliver ‘just-in-time’ interventions. A case example included in the review is the treatment app A-Chess (Johnson et al., 2016), which aims to reduce substance use. By utilizing the smartphone’s GPS, the app automatically triggers and sends intervention messages when users are nearing a high-risk location, such as a bar.

Opportunities for intervention purposes unique to wearables (i.e., electronic devices such as smartwatches and chest straps that measure physiological activity like sleep patterns, heart rate variability, and activity levels) are presented in the scoping review by Bergwerff et al. (2025). The authors underscore wearables’ ability to conduct continuous, non-invasive physiological measurements as these measures can both assess and predict challenging behavior and mental health. As a case example, Bergwerff et al. (2025) describe a wristband that measures blood volume pulse, electrodermal activity, and motion activity. Based on this data, the wristband could predict aggressive behavior three minutes before it actually occurred (Imbiriba et al., 2023), providing useful information and insight for assessment and treatment.

### Technology and Ethical Considerations

Besides the advantages and opportunities technology offers for the assessment and treatment of justice-involved youth, there are some potential disadvantages and risks that are important to consider. Concerns raised include privacy, confidentiality, and security issues related to online (personal) data and the risk of data breaches. Concerns also include quality-related issues, such as the technical competence required of both clinicians and patients (Stoll et al., 2020). Specifically regarding the juvenile justice setting, this special issue includes a paper by Kip et al. (2025) that discusses ethical challenges related to the use of technology for behavioral and mental health assessment and treatment. They raise ethical points regarding informed consent, reliability and validity, privacy and data security, equity and accessibility, unwanted side-effects, persuasiveness, and evidence-based treatments.

## Conclusion

The empirical papers, scoping reviews, and viewpoint paper that together compose this special issue illustrate some of the potential that technology carries for behavioral and mental health assessment and treatment of justice-involved youth. Technology can support existing assessment and treatment methods, enhancing efficiency and saving time for clinicians. It can also bring innovative methods by creating opportunities that are difficult, unethical, or even impossible to achieve with conventional methods.

The various contributions in the volume also highlight what is still lacking. Remarkably, despite ample examples of technology use in mental health care, the scoping reviews showed that its application is nearly absent in juvenile justice settings. This is regrettable, as this group may particularly benefit from technology because they are digital natives and because of the possibilities technology provides, such as personalization and virtual transportation. It is important to support this vulnerable group with state-of-the-art assessment and treatment methods to ensure the best possible care. Therefore, more research is needed on the best methods to incorporate technology into assessment and treatment and to ensure its added value. To achieve this, we call for collaborations between researchers and the clinical field to conduct technology-focused studies with strong, innovative research designs that are feasible within the juvenile justice setting.

## Data Availability

No data was generated for this paper.
